# Secondary Hyperparathyroidism Presenting as a Brown Tumor: A Case Report and Review of the Literature

**DOI:** 10.7759/cureus.33820

**Published:** 2023-01-16

**Authors:** Rui Flores, Joana Lopes, Sofia Caridade

**Affiliations:** 1 Cardiology, Hospital de Braga, Braga, PRT; 2 Internal Medicine, Hospital de Braga, Braga, PRT

**Keywords:** brown tumor, osteitis fibrosa cystica, renal osteodystrophy, secondary hyperparathyroidism, chronic kidney disease (ckd)

## Abstract

Severe secondary hyperparathyroidism in advanced stages of chronic kidney disease (CKD) is associated with CKD-related mineral and bone disease (CKD-MBD). A 70-year-old woman was admitted at the Hospital for generalized bone pain and peripheral edema. Initial laboratory tests revealed normocytic anemia and severe renal dysfunction, and further tests evidenced severe secondary hyperparathyroidism. Full-body computerized tomography showed an osteolytic lesion in the right iliac wing. The iliac bone lesion was biopsied and histological examination was consistent with the diagnosis of a brown tumor of hyperparathyroidism.

Brown tumors are a rare variant of osteitis fibrosa cystica that results from sustained high levels of parathyroid hormone in CKD. This case sheds light on rare complications that are experienced today in CKD. The clinical and biochemical setting, as well as the clinical suspicion, are essential to the diagnosis.

## Introduction

Secondary hyperparathyroidism (SHPTH) is a frequent condition associated with chronic kidney disease (CKD) and represents a major risk factor for cardiovascular mortality and morbidity [1,2]. SHPTH in advanced stages of chronic kidney disease (CKD) is associated with CKD-related mineral and bone disease (CKD-MBD). SHPTH in advanced stages of CKD is triggered by persistent hyperphosphatemia, lack of renal vitamin D hydroxylation, and slight decreases in serum calcium, and can therefore increase bone turnover and contribute to renal osteodystrophy [3-5]. Renal failure can promote mineral disarrangements and bone disease, which are responsible for bone pain, deformity, and fractures [3]. The spectrum of bone disease in CKD hosts both high (i.e., osteitis fibrosa cystica and brown tumors) and low (i.e., osteomalacia; adynamic bone disease) extremes of bone remodeling [5,6]. Parathyroid hormone (PTH) levels help to differentiate this clinical spectrum and may help guide treatment [5].

Brown tumors, also called osteoclastomas, are a rare manifestation of secondary hyperparathyroidism and represent a severe and localized form of osteitis fibrosa cystica with hemorrhagic content [6-9]. They consist of non-neoplastic bone lesions with benign behavior [6-9].

There is a significant lack of information concerning the pathophysiology of renal osteodystrophy and most published data describing brown tumors arises from isolated case reports. To our knowledge, there are few described cases of brown tumors localized inside the pelvis. We report a case of a brown tumor presenting with an osteolytic lesion in the pelvis that prompted an early search for the diagnosis of multiple myeloma. Additionally, we performed a literature review of all cases of brown tumor secondary to CKD with SHPTH specifically affecting the pelvic region until April 2020 through the PubMed database. We used the following keywords: “brown tumor” or “brown tumors” and “secondary hyperparathyroidism” and “pelvis” or “sacrum” or “pelvic girdle”.

## Case presentation

A 70-year-old woman, a native of Portugal living in Australia for the past decade, presented at the emergency department, Hospital of Braga, Portugal, with complaints of pain, redness, and swelling of the wrist and right hand after a traumatic fall at home. There were no previous medical records available as the patient had recently returned to Portugal. She claimed that her past medical history was notable for rheumatoid arthritis for almost 40 years and arterial hypertension. She reported a recent hospitalization in Australia a few months back with a mention of a kidney injury due to drug toxicity, although she did not recall the culprit drug. She was usually medicated with four tablets of furosemide 40 mg daily and took acetaminophen 1000 mg occasionally for osteoarticular pain. She denied drug allergies.

At admission, she complained about generalized bone pain, with great functional limitation, and imbalance. She also mentioned decreased urinary output and increased peripheral edema. She denied exertional dyspnea, orthopnea, paroxysmal nocturnal dyspnea; qualitative changes in urine, namely hematuria or foamy urine; asthenia, fatigue or anorexia; nausea or vomiting; skin changes, namely rash or palpable purpura; low back, flank or hypogastric pain; and respiratory symptoms, such as hemoptysis. She denied fever. She denied consumption of herbal products or recent contrasted imaging tests. She denied a family history of kidney disease or hearing loss.

On physical examination, she had significant peripheral edema, mainly in the facial and periorbital regions, and exuberant edema of the right hand and wrist, with associated redness, pain, and functional disability. Physical examination was otherwise normal.

Laboratory tests revealed a hemoglobin level of 9.9 g/dL (normal range {NR} 12.0 - 15.5 g/dL), mean corpuscular volume 30 pg (NR 27 - 33), white blood cell (WBC) count 16.100/µL (NR 4.000 - 11.000/µL) with 93% neutrophils, blood urea nitrogen 189 mg/dL (NR 7 - 22 mg/dL), creatinine 4.3 mg/dL (NR 0.7 - 1.2 mg/dL), glomerular filtration rate 10 mL/min/1.73 m^2^ [NR  90 mL/min/1.73 m^2^ according to CKD EPI 2021), alkaline phosphatase 248 U/L (NR 45-117 U/L), myoglobin 141 ng/mL (NR 13-71 ng/mL) and C-reactive protein (CRP) 14.80 mg/L (NR < 3 mg/L). Arterial blood gas analysis did not show metabolic acidosis. Punctual urine sample showed slight proteinuria, and the cultural sample was negative. Sodium, potassium, chlorides, lactic dehydrogenase, platelet count, and thyroid stimulating hormone (TSH) were normal.

**Figure 1:**
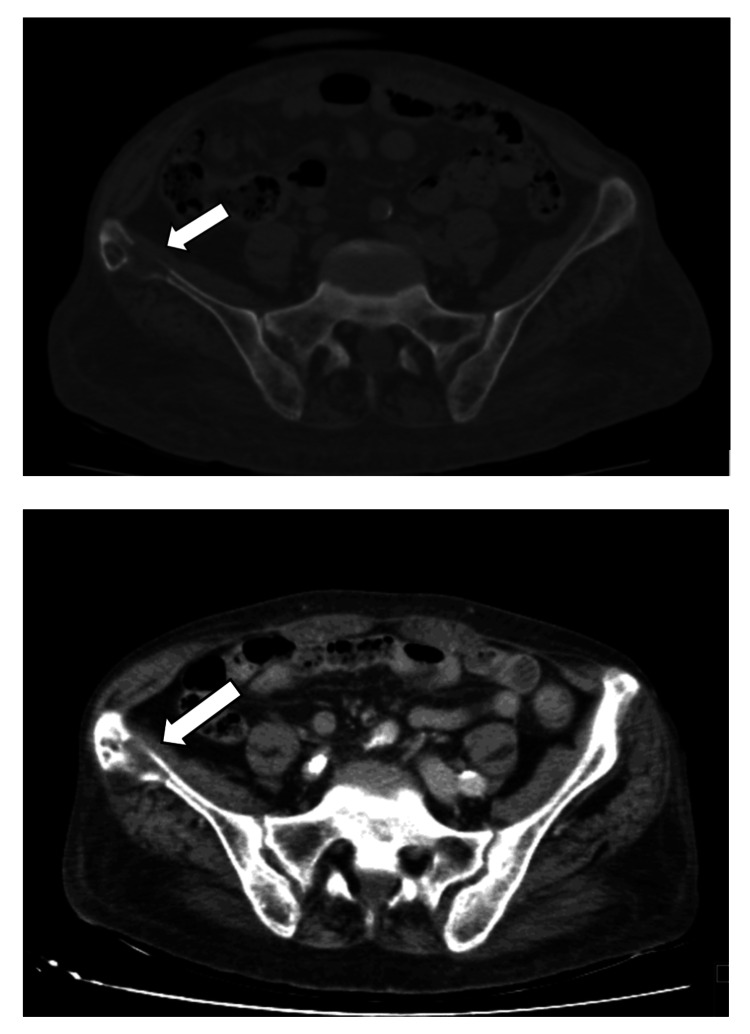
Abdominal CT scan. Transversal views of abdominal CT scan showing an osteolytic lesion in the right iliac wing (arrow), with cortical lysis in its anterior and posterior slopes. No masses in the adjacent soft tissues are visualized.

An abdominal ultrasound (US) revealed normal-sized kidneys with decreased thickness and loss of parenchymal sinus differentiation, alongside multiple bilateral cystic images (maximum length of 12 mm). There were no signs of urinary obstruction. A thoracoabdominopelvic computerized tomography (CT) used to exclude traumatic lesions showed an osteolytic lesion in the right iliac wing (maximum diameter of approximately 25 mm), with evident cortical lysis in its anterior and posterior slopes (Figure 1).

**Figure 2:**
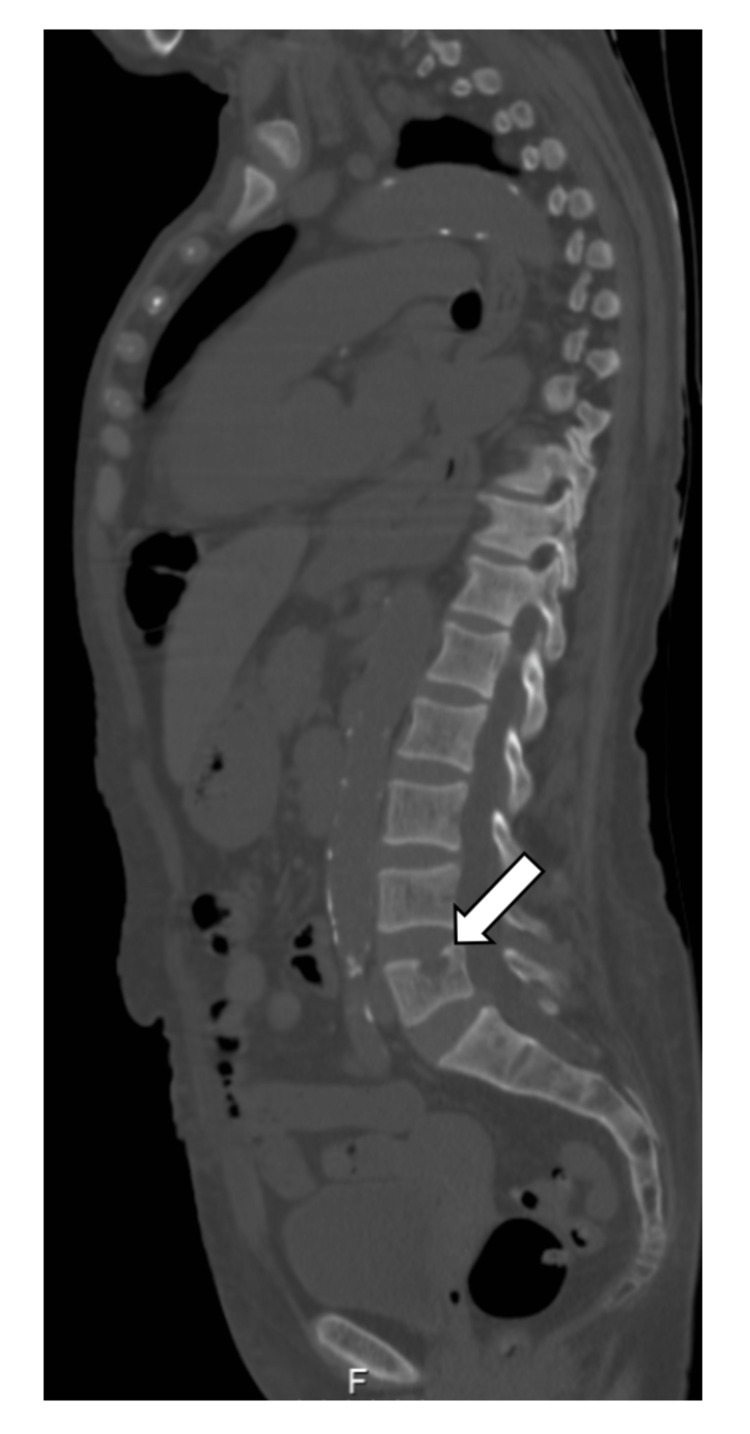
Thoracoabdominopelvic CT scan. Sagittal view of thoracoabdominopelvic CT scan showing a well-defined lytic image on the upper vertebral platform of L5 (arrow) with pericentimetric dimensions.

There were also well-defined lithic images on multiple vertebral platforms (Figure 2).

An isolated and consolidated fracture was seen at the level of the 10th right costal arch. A type I esophageal hernia was also seen. The right hand and wrist did not show any signs of fracture.

Due to anemia, kidney dysfunction, and osteolytic lesions, the patient was admitted for clinical study in the Internal Medicine department. The 24-hour urinary protein excretion was 2.88 g. PTH level was 196 pmol/L (NR 1.96 - 9.3 pmol/L) and vitamin D 15 ng/mL (NR 40 - 80 ng/mL). Serum phosphate and calcium were normal. The erythrocyte sedimentation rate was markedly elevated (108 mm/hr, NR 0 - 29 mm/h). The autoimmunity panel, including anti-nuclear antibodies (ANA), DNA double-stranded antibodies (dsDNAab), complement level, anti-neutrophil cytoplasm antibodies (ANCA), anti-glomerular basement membrane antibody, rheumatoid factor, and cryoglobulins, was unremarkable. Infectious serologies for syphilis, hepatitis B and C, and HIV were negative. Serum protein electrophoresis and protein immunoelectrophoresis were normal, as were the level and ratio of serum and urinary light chains. Immunoglobulin (Ig) counts were normal, except for a slight deficit of IgA (< 8 mg/dL, NR 70 - 400 mg/dL). Iron kinetics, folate, and vitamin B12 levels were also normal. Screening for occult neoplasia, including the pap smear, mammography, and endoscopies of the gastrointestinal tract, did not show any suspicious lesions. The patient’s skin was examined to exclude the possibility of melanoma.

**Figure 3:**
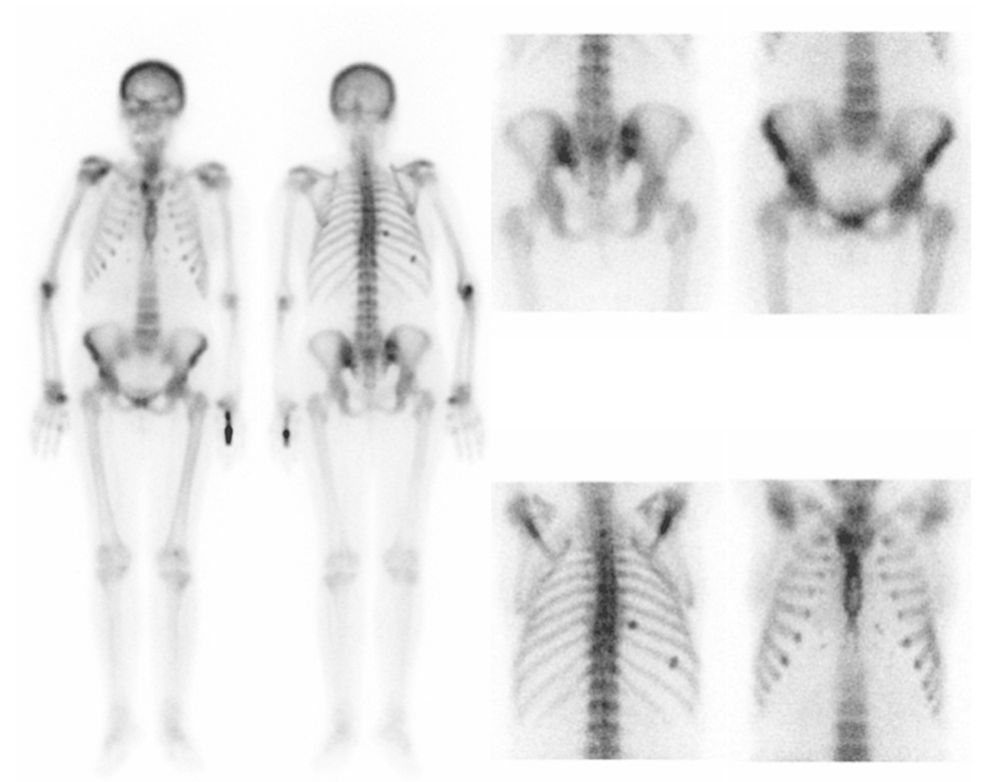
Full-body bone technetium pertechnetate scintigraphy. Full-body bone technetium pertechnetate scintigraphy showing a higher technetium uptake in both shoulders, the 10th right costal arch, several lumbar vertebrae and sacroiliac joints. A diffuse skull cap uptake is also seen.

A full-body bone technetium pertechnetate scintigraphy showed a significantly higher technetium uptake in both shoulders, the 10th right costal arch, several lumbar vertebrae, and sacroiliac joints. A diffuse skull cap uptake was also seen (Figure 3).

The iliac bone lesion was biopsied. Histological examination showed spongy bone tissue partially involved by a fibrous stromal lesion, in which bone trabeculae were surrounded by osteoblasts and a few scattered multinucleated giant cells. These findings allowed the diagnosis of a brown tumor of hyperparathyroidism.

**Figure 4:**
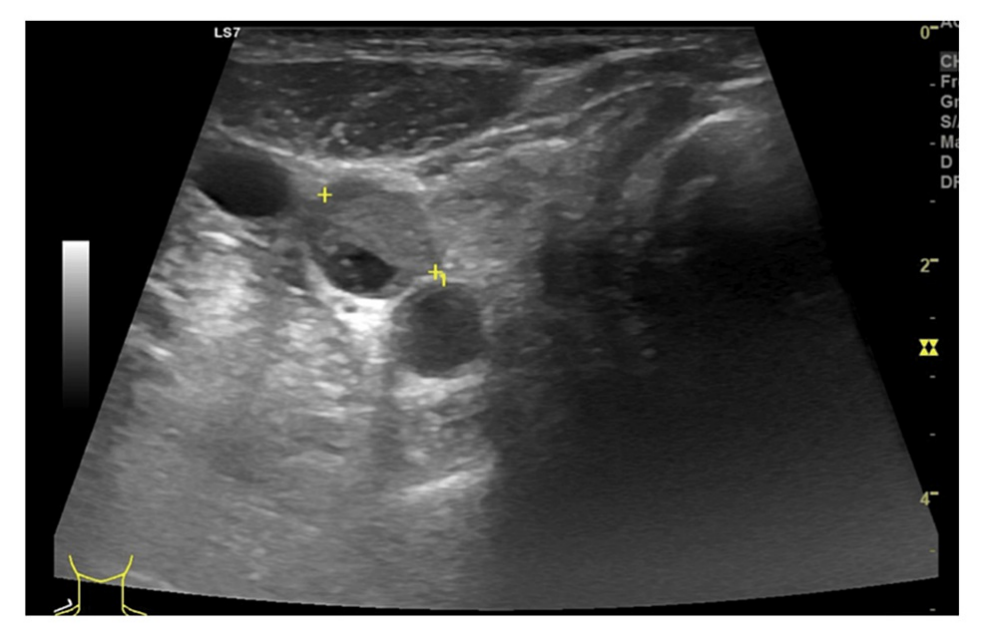
Cervical ultrassound. Cervical US measuring a mixed nodule in the thyroid gland with approximately 17 mm in diameter.

Cervical US revealed a normal-sized thyroid gland with multiple centimetric mixed nodules, the largest of 17 mm that was positioned on the right hemithyroid (Figure 4).

Scintigraphy with technetium sestamibi showed an irregular and slightly heterogeneous thyroid gland but did not show any abnormal uptake or retention of the contrast. The diagnosis of severe secondary hyperparathyroidism with high bone remodeling disease was formulated and the patient was discharged with paricalcitol 2 mcg daily.

The follow-up visit was set to occur about six months after discharge, but three months after discharge the patient developed uremic symptoms that implicated an early start of definitive renal replacement therapy. Clinical response for calcimimetics was suboptimal and referral to parathyroidectomy was made, which was ultimately refused by the patient.

## Discussion

The brown tumor is an enigmatic clinical entity that became rare after the beginning of the 21st century because of the early diagnosis of CKD and hyperparathyroidism [9]. Renal replacement therapies, such as hemodialysis and peritoneal dialysis, have reduced the incidence of severe forms of SHPTH and abnormal bone mineralization [9,10]. The incidence of brown tumors is historically higher in cases of primary hyperparathyroidism, although the incidence in cases of SHPTH is not negligible [10].

Brown tumors are a variant of osteitis fibrosa cystica that results from sustained high levels of PTH and high rates of bone reabsorption [8,10,11]. They are either isolated or multiple osteolytic lesions, with progressive growth, cystic morphology, and the ability to shape and erode the cortical bone without the major involvement of the adjacent soft tissues. Histologically, multinucleated osteoclast-like giant cells are visible with invasion and destruction of the trabecular bone, together with a stromal component constituted by mononuclear cells. The nomenclature of brown tumors is related to the macroscopic red-brown coloration derived from foci of hemosiderin and secondary hemorrhages [10-13]. Although suggestive, histologic appearance should be taken together with the clinical and biochemical scenario to perform a definitive diagnosis [13].

Most brown tumors affect the axial skeleton, namely the jaw, ribs, clavicles, and pelvis, but they can virtually affect any human bone [6-10,14]. Symptoms are rare but can arise from the compression of surrounding structures [14]. According to Kidney Disease Improving Global Outcomes (KDIGO) 2017, clinical guidelines on prevention and treatment of CKD-associated mineral and bone disorders, serum calcium, phosphorus, alkaline phosphatase, and PTH levels should be regularly evaluated to determine the need for specific measures or therapies, such as limiting dietary phosphate, use of phosphate binders, active vitamin D, calcimimetics, or even renal replacement therapies [15]. These strategies aim to reduce PTH levels and calcium burden, therefore improving quality of life, and cardiovascular, renal, and mortality outcomes [16]. Parathyroidectomy is recommended for refractory cases of SHPTH and represents an effective approach for reducing symptoms, PTH levels and cardiovascular outcomes in patients with CKD [3,4,17]. Unfortunately, other issues arise after surgical treatment, including hungry bone syndrome [4]. Extreme cases may imply surgical removal if they cause neurological deficits or are positioned in a critical site [11]. The use of the calcimimetic cinacalcet significantly decreased the need for more aggressive treatments [3].

In our literature review, we found only four cases of brown tumors secondary to CKD with SHPTH affecting the pelvic region (Table 1). Most patients were men (75%) with a mean age of 42 years. Only one patient (25%) had an isolated involvement of the pubis. All four patients had markedly elevated PTH levels (92.8 - 265.1 mmol/L) and presented with osteoarticular pain and/or neurological deficits. All patients were proposed for parathyroidectomy.

**Table 1:** Up-to-date review of literature cases of brown tumors of the pelvic girdle associated with SHPTH. NS – not specified;

Reference	Age	Sex	Location	Renal primary disease	PTH [mmol/L]	Calcium	Phosphate	Alkaline phosphatase	Symptoms	Treatment
Tarrass et al., 2006 [18]	42	M	Lumbosacral region	Chronic glomerulonephritis	154.4	Normal	Normal	High	Cauda equina syndrome	Wide decompressive laminectomy; subtotal parathyroidectomy.
Karagoz et al., 2013 [11]	40	F	Sacrum	NS	92.8	Normal	High	High	Localized pain	Dialysis; radiotherapy; patient declined surgical removal of tumor; proposed to parathyroidectomy.
Araújo et al., 2013 [19]	47	M	Lumbosacral region	Hypertensive nephrosclerosis	265.1	Normal - high	Normal	Normal - high	Osteoarticular pain and paraplegia	Dialysis; percutaneous ethanol injection (parathyroidectomy indicated but not performed due to surgical risk).
Glushko et al., 2015 [8]	39	M	Left pubis	Polycystic kidney disease	259.7	High	High	NS	Fracture after minor trauma	Dialysis; renal transplantation (failed); patient declined surgical approach.

We describe a rare case of pelvic brown tumor attributed to secondary hyperparathyroidism. The patient was admitted with kidney injury of undetermined cause. Following the etiological study, several osteolytic lesions were found. The combination of renal dysfunction, anemia, and osteolytic lesions directed the early search for multiple myeloma. Despite the apparent linear diagnosis, the study of plasma cell dyscrasias was negative. The complementary study for renal injury and the biopsy of the iliac bone lesion was fundamental to establishing the diagnosis of brown tumor. This case sheds light on rare complications that are experienced today in CKD. The clinical and biochemical setting, as well as the clinical suspicion, are essential to the diagnosis.

## Conclusions

Severe complications of CKD are increasingly rare due to the introduction of treatment at an early stage. CKD-MBD, and in particular the brown tumor, is one such possible complication. Brown tumors result from an exaggerated bone demineralization process complicated by hemorrhagic transformation. These injuries affect the appendicular skeleton more and may increase the risk of bone fracture. We report the extremely rare case of a brown tumor in the axial skeleton without adequate response to calcimimetic therapy. The diagnosis of CKD-MBD requires a high rate of clinical suspicion.
